# How to Tell a Horse’s Age

**Published:** 1885-07

**Authors:** 


### How to Tell a Horse's Age.

The foal is born with twelve grinders. When four front teeth have made their appearance, the colt is twelve days old; and when the next four assert themselves its age will be about twenty-eight days. The corner teeth make their appearance when the foal is eight months, and these latter attain the height of the front teeth at the age of a year. The two-year-old has the kernel—The dark substance in the middle of the tooth's crown—ground out of all the front teeth. In the third year the middle front teeth are being shifted; and when three years old those are substituted by the permanent or horse teeth, which are larger and more yellow than their predecessors. The next four teeth are shifted in the fourth year, and the corner teeth in the fifth, giving place to the permanent nipper.

At five years of age a horse has forty teeth, of which twenty-four are grinders far back in the jaw, with which we have little to do.

But, be it remembered, horses invaribly have tushes, which mares very rarely do. Before the age of six is arrived at, the tush is full grown, and has a slight groove on its internal surface (which generally disappears with age, the tush itself becoming more rounded and blunt) ; and at six the kernel or mark is worn out of the middle front teeth. There will still be a difference of color in the center of the tooth.

The tushes have now attained their full growth, being nearly or quite an inch in length ; convex without, concave within, tending to a point, and the extremity somewhat curved. Now, or perhaps some months before, the horse may be said to have a perfect mouth.

At seven years, the mark, as described, is very nearly worn out of the four center nippers, and fast wearing away in the corner teeth —especially in mares ; but the black mark still remains in the center of the tooth, and is not completely filled up until the animal is eight years old. As he gets on past seven the bridle teeth begin to wear away.

At eight the kernel has entirely disappeared from all the lower nippers, and begins to decrease in the middle nippers. It is now said to be "past mark of mouth."

When more than seven, the knowing ones are accustomed to go by appearance of the upper fronts, from which some conclusion may certainly be drawn, as the marks remain in them long after they have been lost from the bottom ones. Much reliance can never be placed on the tushes ; for sometimes they may be found quite blunt at eight, and as often remain pointed at eighteen, and sometimes those in the same mouth will show an apparent difference of a year or more.

There are indications which enable very shrewd observers to guess at a horse's age after eight years even, but none to enable accurate determination. In the ninth year the mark has entirely disappeared from the upper middle teeth, and the hook on the corner only has increased in proportion as the bridle teeth lose their points. At eight the upper surfaces of the nippers are all oval, and as the animals get older they diminish in width, but not in thickness ; they become more rounded and appear wider apart.

At twelve years of age the crown of all the lower front teeth has become somewhat triangular, and the bridle teeth much worn down ; but anything further must be left to experts, and would serve no useful purpose to enlarge upon here. We must not, however, omit to mention the fact that as horses advance in age, their gums shrink away, conveying that long, narrow appearance of the teeth which has long formed the subject of a proverb. They likewise lose their upright appearance, and appear to lean forward, more particularly the upper ones, which assume an arched shape.

Beyond the indications of age afforded by the teeth are some others, which very little experience will render familiar. A dark colored horse—as a brown or a bay—will, in time, turn gray about the face, mane and top of the tail. The back becomes hollow, and the pit or cavity about the eyes gets, by degrees, more and more pronounced.—*Dublin Farmer's Gazette.*

